# Comparing agronomic and phenotypic plant characteristics between single and stacked events in soybean, maize, and cotton

**DOI:** 10.1371/journal.pone.0231733

**Published:** 2020-04-27

**Authors:** Marcia Jose, Hallison Vertuan, Daniel Soares, Daniel Sordi, Luiz F. Bellini, Rafael Kotsubo, Geraldo U. Berger

**Affiliations:** Regulatory Science, Bayer Crop Science., São Paulo, SP, Brazil; USDA-ARS Southern Regional Research Center, UNITED STATES

## Abstract

Genetically modified (GM) crops are one of the most valuable tools of modern biotechnology that secure yield potential needed to sustain the global agricultural demands for food, feed, fiber, and energy. Crossing single GM events through conventional breeding has proven to be an effective way to pyramid GM traits from individual events and increase yield protection in the resulting combined products. Even though years of research and commercialization of GM crops show that these organisms are safe and raise no additional biosafety concerns, some regulatory agencies still require risk assessments for these products. We sought out to investigate whether stacking single GM events would have a significant impact on agronomic and phenotypic plant characteristics in soybean, maize, and cotton. Several replicated field trials designed as randomized complete blocks were conducted by Monsanto Regulatory Department from 2008 to 2017 in field sites representative of cultivation regions in Brazil. In total, twenty-one single and stacked GM materials currently approved for in-country commercial use were grown with the corresponding conventional counterparts and commercially available GM/non-GM references. The generated data were presented to the Brazilian regulatory agency CTNBio (National Biosafety Technical Committee) over the years to request regulatory approvals for the single and stacked products, in compliance with the existing normatives. Data was submitted to analysis of variance and differences between GM and control materials were assessed using *t*-test with a 5% significance level. Data indicated the predominance of similarities and neglectable differences between single and stacked GM crops when compared to conventional counterpart. Our results support the conclusion that combining GM events through conventional breeding does not alter agronomic or phenotypic plant characteristics in these stacked crops. This is compatible with a growing weight of evidence that indicates this long-adopted strategy does not increase the risks associated with GM materials. It also provides evidence to support the review and modernization of the existing regulatory normatives to no longer require additional risk assessments of GM stacks comprised of previously approved single events for biotechnology-derived crops. The data analyzed confirms that the risk assessment of the individual events is sufficient to demonstrate the safety of the stacked products, which deliver significant benefits to growers and to the environment.

## Introduction

The selection of better agronomic plant characteristics by humankind dates back to at least 10,000 years [1]. Throughout millennia, conventional breeding has played a pivotal role in generating the variability of currently domesticated species. More recently, Biotechnology has been harnessed as a molecular tool for the specific introduction of genes of interest in several organisms, including plants used as agricultural crops. Hence, additional characteristics that could not have been obtained as efficiently through classical plant breeding have become available to growers worldwide [2–5]. In this way, genetically modified (GM) crops providing agronomic traits such as herbicide tolerance (HT) and insect protection (IP) obtained early commercial success and were of paramount importance for the much-needed yield gains and environmental benefits [6]. Stacked products combining two or more single GM events were soon after developed by conventional breeding, providing a convenient and successful way to associate distinct characteristics to improve farmer’s flexibility and crop performance allowing them to meet their needs under complex farming conditions [2,3,5]. This resulted in high acceptance and utilization by farmers, and by the end of 2017 stacked GM soybean, maize, and cotton products accounted for 41% of the total global area planted with biotech crops, representing a 3% increment over the previous year–the highest year-to-year growth in the history of modern biotechnology [6].

GM soybean, maize, and cotton have considerably favored the global economy. Between 1996 and 2016 biotech crops commercialization rendered a total farm income gain of US$ 186.1 billion [6]. Small farmers in developing countries are among the most benefited, having earned US$ 5.06 for every dollar invested in biotech crop seeds in 2016, vs. US$ 2.70 for farmers in developed countries [6]. GM crops are indeed credited to result in increased yields with a vast and ample record of safety corroborated by the reviews and approvals of many regulatory agencies across the globe. Finally, the adoption of GM crops brought environmental benefits associated with optimized use of pesticides and fuel reduction and facilitated conservation tillage practices [6,7].

The extensive scientific research conducted to be presented prior to commercialization serve as the basis for assessing the risk and for the commercial approval of products by the designated in-country regulatory agencies. Among the several data types used for risk assessment are agronomic characteristics and plant emergence (considered to relate to the potential for the plant to volunteer in subsequent crops) which are part of the Environmental Risk Assessment (ERA) spectrum [8–11].

Those data, after rigorous science-based regulatory reviews, led to the approval of dozens of GM crops currently commercialized worldwide [12]. Among those crops are stacked GM products, which are also required to undergo regulatory studies in some countries despite being generated by conventional breeding. The current regulatory framework considers it essential to assess the risks of stacked products compared to conventional (non-GM) counterparts or commercially available GM/non-GM references in order to gain commercial approval. This is despite numerous publications showing similarity to conventional controls or corresponding single-events products in compositional profile [13–18], transgene product levels [19], lack of impact on non-target organisms [20,21], and agronomic performance [11,22,23]. These studies have led to very similar conclusions regarding risk concerns, demonstrating that the combined GM products are no different than either of the single GM events, and not substantially different from conventional comparator/control materials concerning any of the analyzed endpoints.

Here we report results from agronomic and phenotypic plant assessments for several soybean, maize, and cotton GM stacked products grown in multiple replicated fields in the northern and southern agricultural regions of Brazil. These results, along with several other data sets, are part of official documents compiled to support regulatory submissions for all types of use of the referred biotechnology-derived crops in Brazil, all of which were granted approval by Brazil’s National Biosafety Technical Committee (CTNBio).

## Materials and methods

### Field trials

All studies were conducted with prior authorization from CTNBio (Comissão Técnica Nacional de Biossegurança), the National Technical Commission on Biosafety, the local federal regulatory body responsible for handling matters related to genetically modified organisms (GMOs), including the GM crops that were under regulated status at the time the studies were conducted. Soybean, maize, and cotton trials were conducted in locations representative of agricultural regions in Brazil (Table 1). This study considered several agronomic characteristics for soybean (initial stand, final stand, days to 50% flowering, lodging, plant height, yield, and 1000 grain weight), maize (initial stand, final stand, days to 50% pollen shed, stay green, ear height, plant height, yield, and 1000 grain weight), and cotton (initial stand, final stand, days to 50% flowering, plant height, days to maturity, and yield). Clarifying some characteristics, stay green in corn is characterized by a longer green state of the plant in the late period of grain filling and days to maturity in cotton was assessed when 60% of open bolls were observed. The potential for volunteering was assessed through percentages of plant emergence. All experiments were arranged as randomized complete blocks with four repetitions in replicated field trials spanning from 2008 to 2017, and comparisons were performed between single or stacked GM materials (Table 2) and the respective non-GM counterparts used as controls (with the same genetic background). Commercial GM and non-GM references were included at each site. The data from the references were indicative of variability that may already occur in each crop for each characteristic considered. All experiments were conducted under good agricultural practices that promoted experimental quality and data integrity. Comparative characterization between data obtained from GM and conventional materials considered the essential aspects of the principle of substantial equivalence, implying that apart from the desired GM-induced effect, all other characteristics should not be altered beyond a known, acceptable variation [24].

**Table 1:** Field trial locations and associated characteristics.

Sites	State; Region	Altitude (m)	Latitude; Longitude	Climate	Soil
Não-Me-Toque	Rio Grande do Sul; South	500	28°24’20” S; 52°48’27" W	Subtropical	Dystrophic red latosol
Rolândia	Paraná; South	600	23°16’30" S; 51°19’45" W	Tropical (Central Brazil)	Red latosol
Santa Cruz das Palmeiras	São Paulo; Southeast	650	21°49’36" S; 47°15’03" W	Tropical (Central Brazil)	Red latosol
Cachoeira Dourada	Minas Gerais; Southeast	450	18°36'58" S; 49°26'21" W	Tropical (Central Brazil)	Red latosol
Luís Eduardo Magalhães	Bahia; Northeast	825	12°07’26” S; 46°01'55" W	Tropical	Entisol
Sorriso	Mato Grosso; Central-West	360	11°43’38” S; 55°06'36" W	Tropical	Yellow red latosol

**Table 2:** Single and stacked products assessed in field trials for agronomic and phenotypic plant characteristics.

Crop	Single/stacked products	Trait	Corresponding transgenic gene product
**Soybean**	MON 87701	Insect resistance	IR: Cry1Ac
	MON 89788	Herbicide tolerance	HT: CP4 EPSPS
	MON 87708	Herbicide tolerance	HT: DMO
	MON 87751	Insect resistance	IR: Cry1A.105, Cry2Ab2
	MON 87701 × MON 89788 (Soybean stack 1)	Insect resistance and herbicide tolerance	IR: Cry1Ac HT: CP4 EPSPS
	MON 87708 × MON 89788 (Soybean stack 2)	Herbicide tolerance	HT: DMO, CP4 EPSPS
	MON 87751 × MON 87708 × MON 87701 × MON 89788 (Soybean stack 3)	Insect resistance and herbicide tolerance	IR: Cry1A.105, Cry2Ab2, Cry1Ac HT: DMO, CP4 EPSPS
**Maize**	MON 87427	Herbicide tolerance	HT: CP4 EPSPS
	MON 89034	Insect resistance	IR: Cry1A.105, Cry2Ab2
	MON 87411	Insect resistance and herbicide tolerance	IR: Cry3Bb1, *DvSnf7* HT: CP4 EPSPS
	MON 88017	Insect resistance and herbicide tolerance	IR: Cry3Bb1 HT: CP4 EPSPS
	MON 89034 × MON 88017 (Maize stack 1)	Insect resistance and herbicide tolerance	IR: Cry1A.105, Cry2Ab2, Cry3Bb1 HT: CP4 EPSPS
	MON 87427 × MON 89034 × MIR162 × MON 87411 (Maize stack 2)	Insect resistance and herbicide tolerance	IR: Cry1A.105, Cry2Ab2, Vip3Aa20, Cry3Bb1, *DvSnf7* HT: CP4 EPSPS
**Cotton**	MON 15985	Insect resistance	IR: Cry1Ac, Cry2Ab2
	MON 88913	Herbicide tolerance	HT: CP4 EPSPS
	MON 15985 × MON 88913 (Cotton stack 1)	Insect resistance and herbicide tolerance	IR: Cry1Ac, Cry2Ab2 HT: CP4 EPSPS
	MON 88913 × MON 88701 (Cotton stack 2)	Herbicide tolerance	HT: DMO, CP4 EPSPS, PAT
	COT102 × MON 15985 × MON 88913 (Cotton stack 3)	Insect resistance and herbicide tolerance	IR: Vip3Aa19, Cry1Ac, Cry2Ab2 HT: CP4 EPSPS
	COT102 × MON 15985 × MON 88913 × MON 88701 (Cotton stack 4)	Insect resistance and herbicide tolerance	IR: Vip3Aa19, Cry1Ac, Cry2Ab2 HT: CP4 EPSPS, DMO, PAT

Products are indicated by their event codes. Each biotechnology-derived trait is indicated per single or stacked product, as well as corresponding transgenic gene product (IR: insect resistance; HT: herbicide tolerance). CP4 EPSPS: *Agrobacterium* tumefaciens (strain CP4) 5-enolpyruvylshikimate-3-phosphate synthase (tolerance to glyphosate herbicide); Cry (various proteins): *Bacillus thuringiensis* (different strains) Cry δ-endotoxins (resistance to Lepidopteran/Coleopteran insects); DMO: *Stenotrophomonas maltophilia* (strain DI-6) dicamba mono-oxygenase (tolerance to dicamba herbicide); *DVSnf7*: *Diabrotica virgifera virgifera* double-stranded RNA transcript containing a 240 bp fragment of the *Snf7* gene (resistance to Coleopteran insects); PAT: *Streptomyces hygrosopicus* phosphinothricin N-acetyltransferase (tolerance to glufosinate herbicide); Vip3Aa19/Vip3Aa20: *Bacillus thuringiensis* (strain AB88) vegetative insecticidal protein (Lepidopteran insect resistance). All gene products are proteins, except for the double-stranded RNA molecule *DvSnf7*.

### Data and statistical analyses

Combined-field site analyses between GM and control materials were performed. For the combined-field site analysis, soybean materials were divided into maturity groups (MG) 5 (South states) and 8 (North states); maize materials were grouped as one using the same germplasm or divided according to South and North Region-specific germplasms to assess plant volunteer of maize stack 1 and single; cotton materials were all grouped as one.

Analysis of variance was based on the randomized block design and differences in mean values for test and control materials were assessed by t-test at the 5% level of significance. Reference ranges were determined from the minimum and maximum mean values observed among the reference materials, where each mean was combined over all sites at which the reference was planted. For agronomic characteristics evaluation data was normalized to control (expressed as the ratio of the GE product value to the control value); for volunteer plant evaluation data was expressed as the percentage of emerged plants (number of emerged plants/number of planted seeds × 100%).

## Results

### Soybean

For soybean, initial and final stand, days to 50% flowering, lodging, plant height, grain yield, and 1000 grain weight were the selected agronomic attributes. Field trial results indicated the great majority of agronomic characteristics for singles and stacks did not differ from the corresponding conventional controls for both MG5 and MG8. Eventual differences were within the commercial reference ranges, not reproducible between MGs, and/or did not impact additional agricultural aspects nor were biologically relevant.

**Fig 1:**
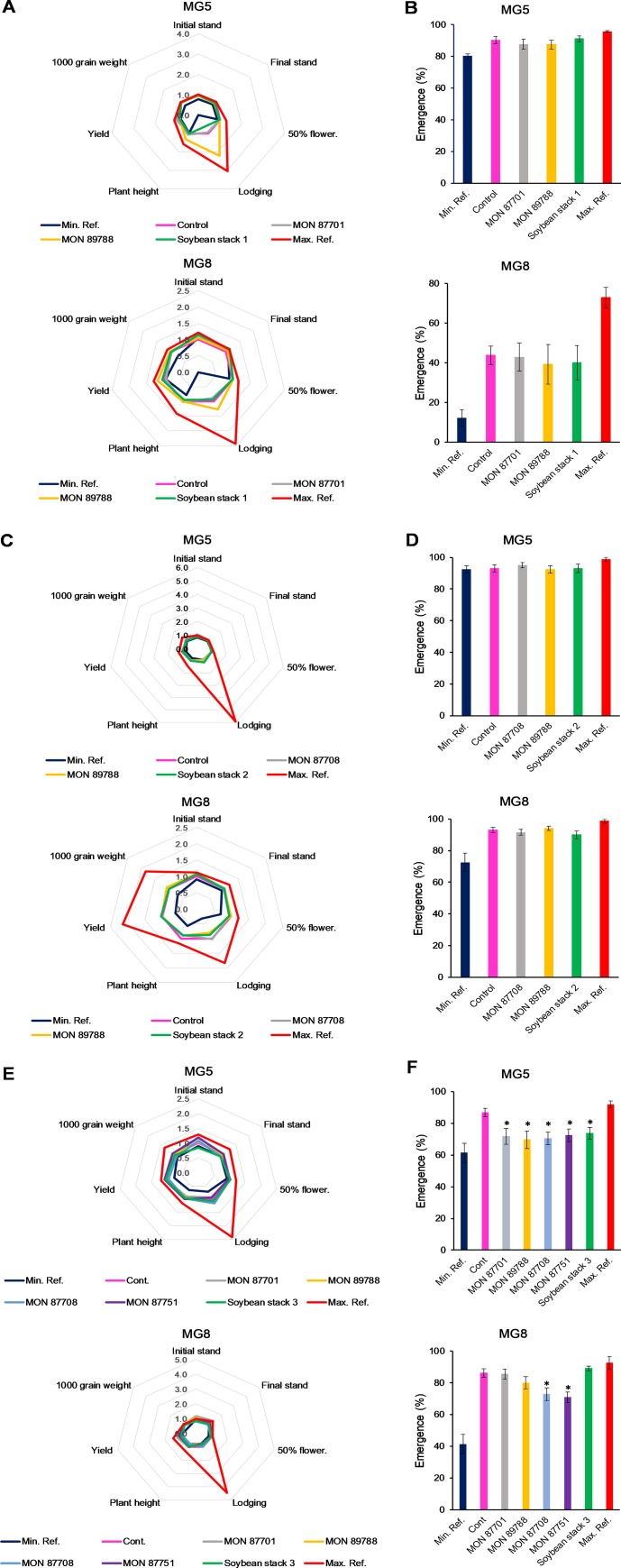
Agronomic and volunteer plant evaluation in soybean stacks 1–3 and single events. Combined (stacked), single, conventional (control), and commercial reference materials were analyzed for agronomic (A, C, and E) and volunteer plant characteristics (B, D, and F). 50% flower = days to 50% flowering; Yield = grain yield. Test materials (stacked or single GM materials) were compared to controls (conventional counterparts) and commercially available reference materials providing minimum and maximum mean values used to create a crop-specific, representative comparison interval. Data was normalized to control (A, C, and E) or as percentage of emerged plants (B, D, and F) for graphic representation. Analysis of variance (t test) was performed based on the experimental randomized block design. Statistical significance at *p* ≤ 0.05. Locations: 4 sites for Soybean stack 1 and its singles (Não-Me-Toque, Rolândia, Cachoeira Dourada, Sorriso), 6 sites for Soybean stack 2 and Soybean stack 3 and its singles (Não-Me-Toque, Rolândia, Santa Cruz das Palmeiras, Cachoeira Dourada, Luís Eduardo Magalhães and Sorriso).

Soybean stack 1 and its single events MON 87701 and MON 89788 were statistically compared to their control counterpart. Among forty-two comparisons done for MG5 and MG8, thirty-five showed no significant difference (Fig 1A). The seven differences that were found (initial stand, lodging and plant height for MON 87701—MG5, initial and final stand for MON 87701 –MG8 and initial and final stand for soybean stack 1 –MG8) were within the reference range showing that the observed differences are within variation that already occurs in soybean. Volunteer plant analyses showed no differences between GM stacks and single traits when compared to control for either MG5 or MG8 (Fig 1B).

The same was observed for single products MON 87708 and MON 89788 and soybean stack 2 (MON 87708 × MON 89788) when they were analyzed. For the three products, in both growth regions and corresponding MGs, most of analyzed parameters were again not different from the conventional controls (Fig 1C and 1D), including emergence rates. Also, for these products, forty-two comparisons were conducted and thirty-nine of these comparisons (93%) showed no significant difference between the GM products and their conventional control. The exception was initial stand count in MG8: measurements collected from soybean stack 2 and both singles provided a superior mean values when compared to control, all again within the reference limits (Fig 1C, lower panel).

Finally, we sought out to investigate soybean stack 3 (MON 87751 × MON 87708 × MON 87701 × MON 89788) and singles MON 87751, MON 87708, MON 87701 and MON 89788 (Fig 1E and 1F). Again, assessed attributes were predominantly similar between the conventional controls and stacks or singles, and found to be within the reference range when significant differences occurred.

### Maize

**Fig 2:**
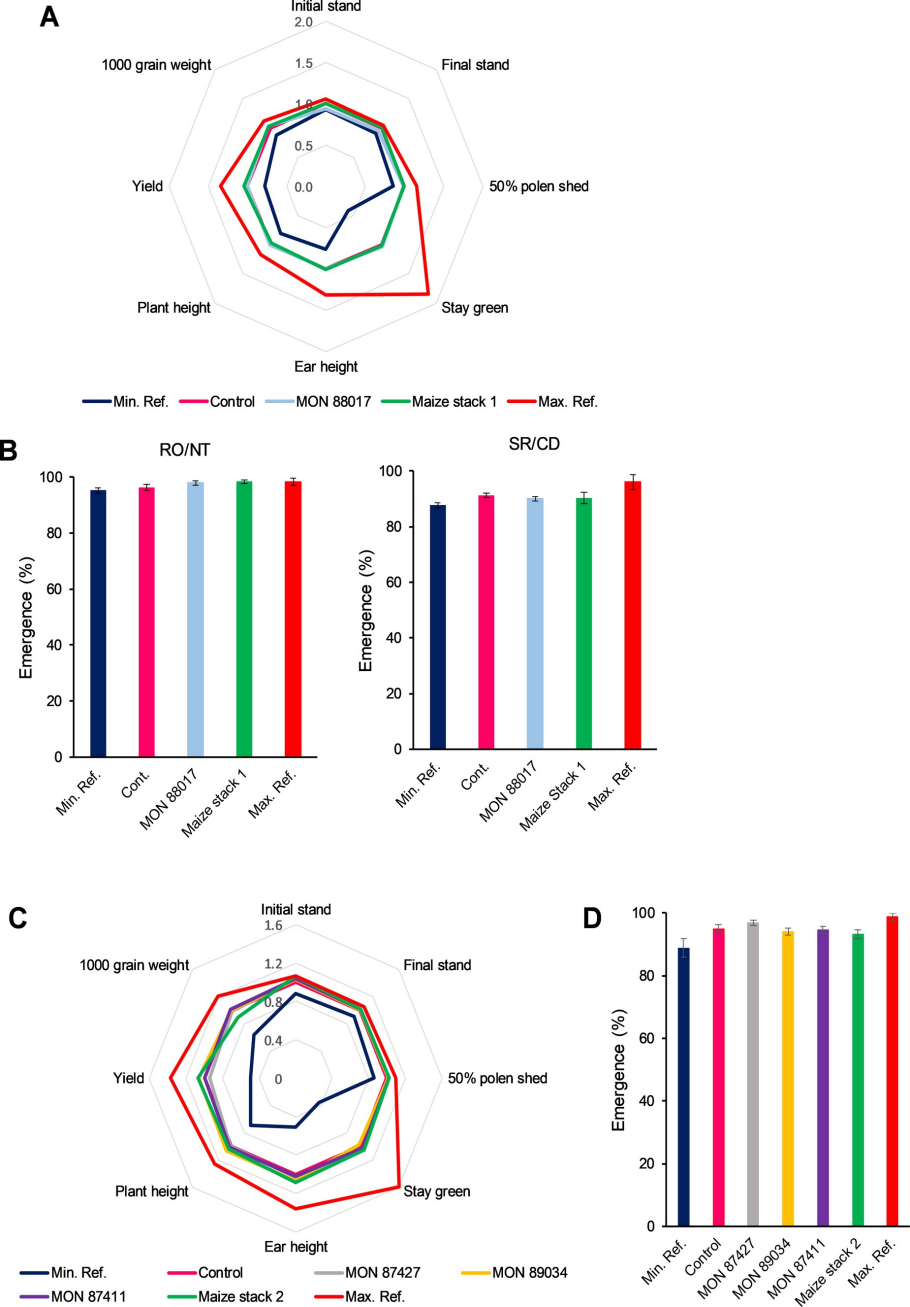
Agronomic and volunteer plant evaluation in maize stacks 1–2 and single events. Combined (stacked), single, conventional (control), and commercial reference materials were analyzed for agronomic (A and C) and volunteer plant characteristics (B and D). 50% pollen shed = days to 50% pollen shed; Yield = grain yield. Test materials (stacked or single GM materials were compared to controls (conventional counterparts) and commercially available reference materials provided minimum and maximum mean values used to create a crop-specific, representative comparison interval. Data was normalized to control (A and C) or as percentage of emerged plants (B and D) for graphic representation. Analysis of variance (t test) was performed based on the experimental randomized block design. Statistical significance at p ≤ 0.05. Locations: 4 sites for Maize stack 1 and MON 88017 (Não-Me-Toque, Rolândia, Cachoeira Dourada, Sorriso) and 6 sites for Maize stack 2 and its singles (Não-Me-Toque, Rolândia, Santa Cruz das Palmeiras, Cachoeira Dourada, Luís Eduardo Magalhães and Sorriso).

Maize stack 1 (MON 89034 × MON 88017) and MON 88017 were evaluated for both agronomic and volunteer plant evaluations. The comparisons between GM crops and the corresponding conventional materials revealed no significant differences (Fig 2A–2D). The only exception was MON 88017 final stand count and 1000 grain weight, which presented, respectively, lower and higher mean values when compared to control. However, both were within the reference range (Fig 2A).

A total of thirty-two statistical comparisons were conducted for maize stack 2 (MON 87427 × MON 89034 × MIR162 × MON 87411) and single products MON 87427, MON 89034, and MON 87411 to the non-GM conventional counterparts in a single experiment (Fig 2C). The majority of those 32 comparisons showed no significant differences between the GM products and conventional controls. Six differences found on these comparisons presented values within reference interval. Emergence values used as an indicator of volunteer plant characteristic indicated no differences between maize stack 2 and single events when compared to the conventional control (Fig 2D).

### Cotton

**Fig 3:**
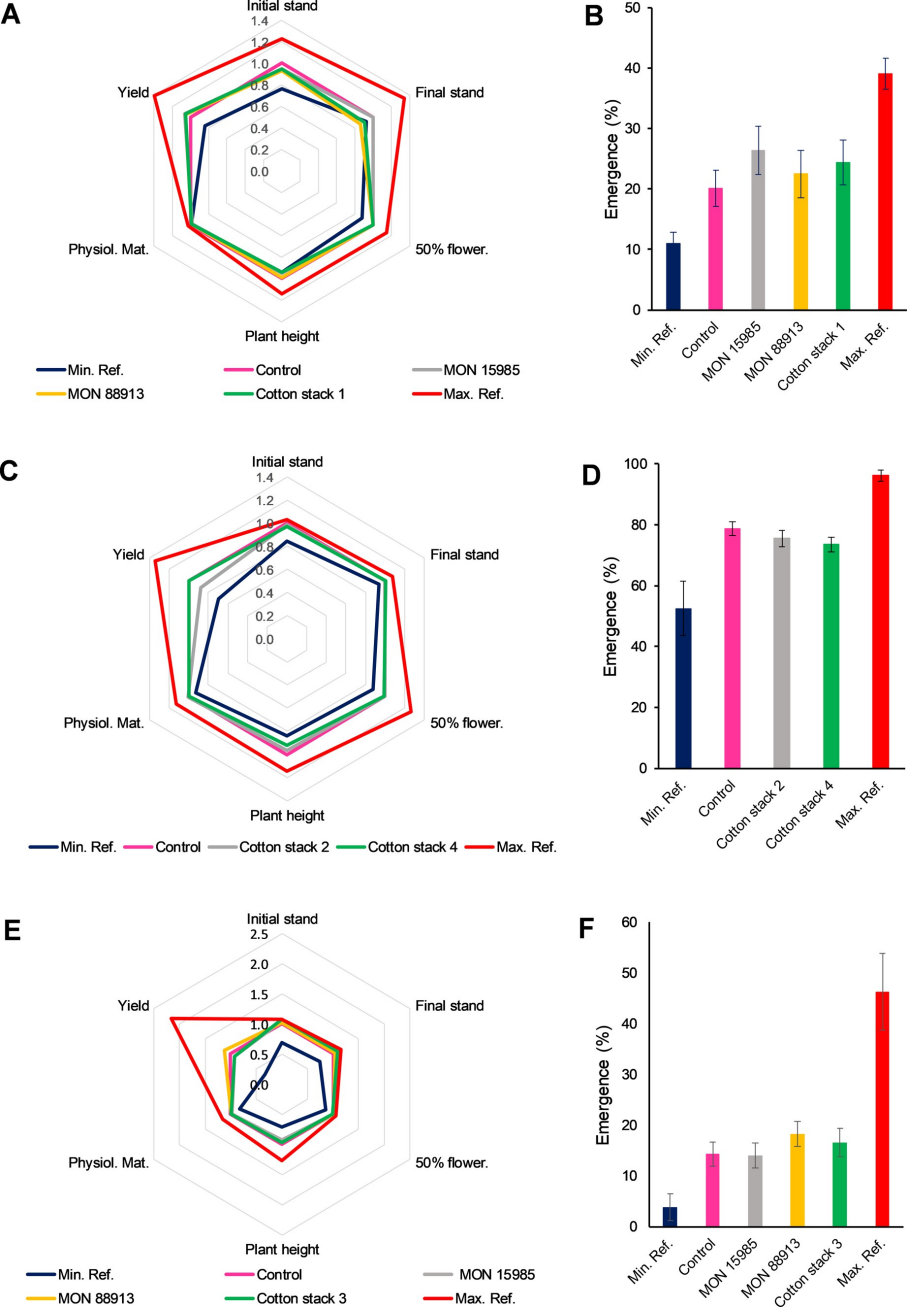
Agronomic and volunteer plant evaluation in cotton stacks 1–4 and single events. Combined (stacked), single, conventional (control), and commercial reference materials were analyzed for agronomic (A, C, and E) and volunteer plant characteristics (B, D, and F). 50% flower. = days to 50% flowering; Physiol. Mat. = physiological maturation; Yield = seed cotton yield. Test materials (stacked or single GE materials) were compared to controls (conventional counterparts) and commercially available reference materials provided minimum and maximum mean values used to create a crop-specific, representative comparison interval. Data was normalized to control (A, C, and D) or as percentage of emerged plants (B, D, and F) for graphic representation. Analysis of variance (t test) was performed based on the experimental randomized block design. Statistical significance at p ≤ 0.05. Locations: 2 sites for Cotton stack 1 and its singles (Cachoeira Dourada and Sorriso), 5 sites for Cotton stack 2, Cotton stack 3 and Cotton stack 4 (Rolândia, Santa Cruz das Palmeiras, Cachoeira Dourada, Luís Eduardo Magalhães and Sorriso).

Evaluation of the aforementioned agronomic attributes and plant emergence values provided no significant differences between cotton stack 1 (MON 15985 × MON 88913), MON 15985, or MON 88913 *versus* the conventional control (Fig 3A and 3B) in all eighteen comparisons done.

Statistical analysis for cotton stack 2 (MON 88913 × MON 88701), cotton stack 4 (COT102 × MON 15985 × MON 88913 × MON 88701) (Fig 3C and 3D), MON 15985, MON 88913 and cotton stack 3 (COT102 × MON 15985 × MON 88913) (Fig 3E and 3F) showed that most of the comparisons conducted (90%) presented no significant differences. All three differences that were detected (plant height for cotton stack 4, initial and final stand counts for cotton stack 3) in 30 comparisons, were within the respective commercial reference ranges (Fig 3C and 3E). All the other parameters analyzed, including plant emergence (Fig 3C, 3D and 3F), showed no differences when compared to the conventional controls.

## Discussion

The development of a single GM crop is an elaborate, resource-demanding process that involves sieving through thousands of candidate genes, transforming and regenerating thousands of plants, and assessing risk at various levels [25]. The final commercial authorization of each product is preceded by intensive scientific data that guarantee quality and safety. Moreover, several regulatory agencies around the world require risk assessments for stacked GM crops derived from conventional breeding of the single events even though they do not represent new transformation events [26–28].

Stacks were a mere possibility when risk assessment requirements were delineated some three decades ago. Throughout the years, several pertinent aspects were clarified and regulatory agencies around the world have accumulated considerable experience and familiarity with conventionally bred GM stacked products. The World Health Organization has recognized for more than 20 years that when plants substantially equivalent to conventional varieties are crossed by classical breeding, it is expected that the resulting product is also substantially equal to the individual events [24]. FAO’s Codex Alimentarius also provides valuable considerations on this regard and has long recognized that once single events have been deemed safe, their combination through classical breeding could be harnessed for introgression into commercial cultivars without the need for additional safety assessments [29]. Regulatory agencies from the United States [30], Canada [31], Australia [32], and China [33] do not request additional regulatory data to approve combined GM events as long as the singles have already been evaluated and proved safe. These historical institutional positionings make it fair to infer that conclusions from risk assessments of single events can be extended to the stacked products that combine them. Such decision-making approaches are also discussed elsewhere [26,34] and are corroborated by the data presented in this paper. Additionally, Brazil’s CTNBio has recently approved four stacked products (two for cotton and two for corn) based on Normative Resolution 5, in which an article states that upon approval of single products, the corresponding stacked products could be approved through a consultation letter without the need for presenting new data on the stack [35].

Different groups have looked at the potential unintended alterations of stacking single transgenic events. Compositional and gene expression studies concluded for the overall equivalence or prediction of the assessed biological parameters when stacks were compared to non-GM materials or singles [17–19]. Cerqueira and colleagues reported a study where agronomic characteristics of GM maize products MON 89034 × TC1507 × NK603 × DAS-40278–9, MON 89034 × TC1507 × NK603, and DAS-40278–9 were evaluated and compared to a non-GM hybrid counterpart control and two commercial non-GM hybrids [22]. The agronomic attributes for all GM products were found to be statistically indistinguishable from the non-GM hybrid counterpart control, most of which fell within the range of the commercial materials included in the study. This mounting evidence indicates that conventional breeding of GM crops does not pose new risk. Therefore, there seems to be a comfortable space for deliberating whether the current mandatory requirements posed by regulatory guidelines for the approval of stacked GM products are indeed providing answers to relevant risk assessment inquires.

Agronomic/phenotypic and volunteer plant evaluations are two commonly used parameters that constitute commercial petitions for ERA by regulatory agencies. This study summarizes data from field trials with 19 different single and stacked GM products from major global crops aiming at evaluating agronomic and volunteer plant characteristics. Our data demonstrates that stacked GM products, similarly to the single parental GM events, are substantially equivalent to the corresponding conventional materials used as controls. Mean values found in this study generally showed no difference from the non-GM counterpart controls or fell within the range established by commercial GM and non-GM references concomitantly grown in these experiments.

Diversifying, rearranging, and combining genes is in the essence of agriculture since its beginning. Conventional breeding was the biotechnological tool that provided humankind with the best means to achieve the most desired agricultural traits over 10,000 years ago. Modern biotechnology is one of the most powerful tools of modern agriculture and has been a major player for the sustainable growth of this sector, providing techniques that result in specific traits with ever-improving genetic modification precision. Our results are aligned with a growing weight of evidence that indicates the combination of GM crops through conventional breeding does not result in alterations and confirms the environmental safety of the resulting stacks. Also, these results confront the existing requirements from some agencies for additional risk assessments for stacks generated from single events regarded previously as safe. This fact is becoming common sense not only among the scientific community, but also different government and regulatory agencies. Moreover, this positive trending scenario is of paramount importance when all the challenges of the value chain are taken into account, especially considering the need to increase productivity to feed a growing populating under conditions of global warming and intensified pest pressure [36,37]. From small scale R&D firms to growers and consumers around the world, several players could benefit from stacked products developed through conventional breeding techniques that demand less resources and time to be generated. A transition in the perception of the actual risk these products place would considerably help overcome agriculture’s biggest challenges while sustaining the same safety aspects that have been proven right for over 20 years of biotechnology adoption.

## Supporting information

S1 Data (XLSX)

S2 Data (XLSX)

S3 Data (XLSX)
